# The effect of leisure-time physical activity on the risk of acute myocardial infarction depending on Body Mass Index: a population-based case-control study

**DOI:** 10.1186/1471-2458-6-296

**Published:** 2006-12-07

**Authors:** Eleonor Fransson, Ulf de Faire, Anders Ahlbom, Christina Reuterwall, Johan Hallqvist, Lars Alfredsson

**Affiliations:** 1 Division of Cardiovascular Epidemiology, Institute of Environmental Medicine, Karolinska Institutet, Box 210, SE-171 77 Stockholm, Sweden; 2 Department of Cardiology, Karolinska Hospital, Stockholm, Sweden; 3 Division of Epidemiology, Institute of Environmental Medicine, Karolinska Institutet, Stockholm, Sweden; 4 Centre of Public Health, Stockholm County Council, Karolinska Hospital, Stockholm, Sweden; 5 Research and Development Unit, Jämtland County Council, Östersund, Sweden; 6 Division of Social Medicine, Department of Public Health Sciences, Karolinska Institutet, Stockholm, Sweden

## Abstract

**Background:**

High body mass index (BMI) and lack of physical activity have been recognized as important risk factors for coronary heart disease. The aim of the present study was to evaluate whether leisure-time physical activity compensates for the increased risk of acute myocardial infarction associated with overweight and obesity.

**Methods:**

Data from the SHEEP (Stockholm Heart Epidemiology Program) study were used. The SHEEP study is a large Swedish population-based case-control study, comprising 1204 male and 550 female cases, and 1538 male and 777 female controls, conducted in Stockholm County, Sweden, during the period 1992–1994. Odds ratios (OR), together with 95 % confidence intervals (95% CI), were calculated using unconditional logistic regression, as estimates of the relative risks.

**Results:**

Regular leisure-time physical activity was associated with a decreased risk of myocardial infarction among lean, normal-weight and overweight subjects, but not among obese subjects. Obese (BMI ≥ 30) and physically active persons had an almost twofold risk of myocardial infarction, compared with normal-weight and sedentary persons (OR 1.85, 95% CI 1.07–3.18). The results were similar for men and women.

**Conclusion:**

While regular leisure-time physical activity seems to provide protection against myocardial infarction among lean, normal-weight and overweight subjects, this does not appear to be the case in obese subjects.

## Background

Both excess body weight and lack of leisure-time physical activity have been identified as important risk factors for cardiovascular mortality, as well as coronary heart disease [1-13]. These two risk factors are also associated with each other [14,15].

Regular physical activity seems to attenuate much of the increased risk associated with overweight or obesity, and furthermore, active obese persons seem to have lower all-cause mortality and CHD morbidity compared with those who are of normal weight but physically inactive [16]. However, this association needs to be further analyzed in various populations that include both men and women.

In a previous study we have reported on the relation between various forms of physical activity and the risk of acute myocardial infarction [17].

The aim of the present study was to evaluate whether the relationship between leisure-time physical activity and risk of acute myocardial infarction is the same among overweight and obese persons, as in normal-weight individuals. Furthermore, we wanted to study whether leisure-time physical activity lowers the risk of myocardial infarction among overweight and obese persons to the same level, or lower, as among normal-weight but physically inactive persons.

## Methods

The data used in the present analysis comes from the Stockholm Heart Epidemiology Program (SHEEP) study. The SHEEP study is a population-based case-control study of first events of acute myocardial infarction. The study base comprised all Swedish citizens living in Stockholm County during 1992–93 (men) and 1992–94 (women), who were 45–70 years of age and free from clinically diagnosed myocardial infarction. Myocardial infarction was defined using criteria set up by the Swedish Association of Cardiologists in 1991. Cases were identified from the coronary and intensive care units at the internal medicine departments at all the emergency hospitals within the Stockholm County area, the hospital discharge register, and through death certificates from the National Register of Death Causes at Statistics Sweden. If the patient died within 28 days of diagnosis, he or she was defined as having suffered from a fatal myocardial infarction.

One control per case was selected randomly from the study base, after stratification for sex, age and hospital catchment area. In case of non-response, another control, who belonged to the study base at the time of the case occurrence, was randomly chosen. This procedure was repeated at most four times. In some cases, the first control answered after a second control subject had been contacted. In those cases, both the first and second control subject was included in the study. Each control candidate was checked for history of myocardial infarction before inclusion.

Cases and controls were asked to fill out an extensive questionnaire on lifestyle factors. For fatal cases, the questionnaires were sent to a close relative, at the earliest six months after the date of death of the case subject. The surviving cases and their controls were also invited to a clinical examination, which was carried out at least three months after the onset of the myocardial infarction for the cases.

In total, 2246 cases and 3206 controls were invited to the SHEEP study. Of the invited subjects, 1754 cases (1204 male and 550 female), and 2315 controls (1538 male and 777 female) answered the questionnaire and were therefore included in this study. This corresponds to a participation rate of 78 % for the cases (84% for non-fatal and 62% for fatal cases) and 72 % for the controls. In general, the participation rate was slightly higher for men than for women. Of the included cases, 968 males and 413 females survived the first 28 days after diagnosis of their myocardial infarction.

The SHEEP study was approved by the Ethics Committee at Karolinska Institutet, Stockholm. A more detailed description of the SHEEP study can be found in the study by Reuterwall, et al [18].

### Leisure-time physical activity/exercise

The respondents were asked to report their average leisure-time physical activity level and exercise during different age intervals in life (15–24, 25–34, 35–44, 45–54, 55–64, 65–69 years of age).

The pre-defined activity levels were: very little physical activity; occasional walks; some exercise now and then; exercise on a regular basis (at least once per week). Exercise was defined as leisure-time physical activity that lasted for at least 30 minutes and made them out of breath. The respondents were asked to include walking or biking to and from work.

In a follow-up question, the persons who exercised on a regular basis were asked to specify how often they were engaged in the different activities. Those who answered that they exercised on a regular basis, but did not answer the following question about frequency were added to the category of individuals who exercised once per week. This was done for 19 cases and 25 controls.

For the analyses, three categories were then constructed: very little exercise and occasional walks; exercise now and then, once per week; exercise two or more times per week. In the analyses, we used the information regarding physical activity in the age interval to which the subject belonged at the time of the case occurrence.

### Body Mass Index (BMI)

BMI was calculated as weight (kg)/height (m)^2^. Data from the clinical examination were used as the primary source of information, available for 88 % of the non-fatal cases and 68 % of the control subjects. If we did not have data from the clinical examination, information on weight and height from the questionnaire was used. In the analyses, BMI were categorized into four groups: lean (BMI < 20.0), normal weight (BMI 20.0–24.9), overweight (BMI 25.0–29.9), and obesity (BMI ≥ 30.0).

### Potential confounding factors

Age (5-year categories), sex and hospital catchment area were considered as confounding factors in all analyses, on account of matching on these factors.

Smoking was defined as current smoking or non-smoking. Current smoking included subjects who reported smoking at the time of inclusion and those who reported giving up smoking less than two years prior to study inclusion.

Socioeconomic status was categorized into eight categories: unskilled and skilled manual workers, low-, middle-, and high-grade non-manual workers, unskilled and skilled self-employed people, and others (housewives, unemployed, etc.).

Alcohol consumption, fiber intake (used as an indicator of dietary habits), physically demanding household tasks, as well as active/sedentary job, were also considered as potential confounding factors.

### Statistical methods

Since the SHEEP study was designed as a frequency-matched case-control study, odds ratios (OR) together with 95 % confidence intervals (CI) were calculated using unconditional logistic regression [19]. The odds ratios were calculated to estimate the relative risk of acute myocardial infarction between those who were exposed or unexposed to the different combinations of BMI and leisure-time physical activity levels.

Analyses including all cases, as well as analyses only including the non-fatal cases, were performed, due to the fact that we had to rely on proxy information regarding the exposure of BMI and leisure-time physical activity for the fatal cases. Analyses including both men and women, as well as separate analyses for men and women, were also conducted.

## Results

Overweight and obesity, being sedentary during leisure time, smoking, and being classified as a blue-collar worker were more common among cases than controls (table 1).

The separate effects of BMI and leisure-time physical activity are shown in table 2. Leanness (BMI < 20) was associated with an increased risk when all cases were included, but this association was weakened when only non-fatal cases were analyzed. Overweight (BMI 25.0–29.9) was associated with 43–57 % increased risk, while obesity (BMI ≥ 30) was associated with a doubled risk, or more, of myocardial infarction (OR from 2.10 to 2.23) when compared with normal-weight persons. Leisure-time physical activity/exercise at least twice per week was associated with a risk reduction of 38–46 % compared with being sedentary. The separate analyses for men and women yielded very similar results.

The combined effect of BMI and leisure-time physical activity on the risk of acute myocardial infarction is shown in table 3. The group of normal-weight (BMI 20.0–24.9) sedentary individuals was used as the reference category. The results were adjusted for age, sex, hospital catchment area, socioeconomic status and smoking. Further adjustment for alcohol consumption, fiber intake, demanding household tasks and active or sedentary job situation, did not change the estimates in any substantial way (data not shown).

An inverse relationship between leisure-time physical activity and myocardial infarction was noted in all BMI groups except for the group of obese (BMI ≥ 30) individuals (p-value for statistical interaction = 0.05).

As compared with the normal-weight but sedentary group, the overweight (BMI 25.0–29.9) physically active group seemed to have a decreased risk of myocardial infarction (OR 0.79, 95% CI 0.59–1.06). On the contrary, the group of obese (BMI ≥ 30) individuals who were physically active at least twice per week had an 85% increased risk of myocardial infarction (OR 1.85, 95% CI 1.07–3.18), as compared with normal-weight but sedentary persons.

The general pattern was the same for men and women. As in the previous analysis, further adjustment for alcohol intake or fiber consumption did not alter the results in any substantial way (data not shown).

When the analyses were based on the non-fatal cases only, the odds ratios for the overweight and obese persons were shifted upwards, as seen in table 4. In this analysis, the overweight and active group had an equal risk of myocardial infarction as compared with the normal-weight but sedentary group (OR 0.97, 95% CI 0.72–1.32), while the physically active but obese group (BMI ≥ 30) had an odds ratio of 2.27 (95% CI 1.30–3.95) (p-value for statistical interaction = 0.06). The inverse gradient between physical activity and myocardial infarction risk was slightly weakened in the groups of lean, normal-weight and overweight individuals, compared with the analysis when all cases were included.

To evaluate the possible effect of reverse causation, we performed additional analyses excluding individuals who had reported a history of stroke, angina pectoris, claudicatio intermittens, or congestive heart failure in the questionnaire. However, the general pattern in the results was unchanged.

## Discussion

In this population-based case-control study we found an inverse relationship between leisure-time physical activity and the risk of acute myocardial infarction in the groups of lean, normal-weight and overweight individuals, but not in the group of obese persons. Overweight individuals (BMI 25.0–29.9) who where physically active twice or more per week during their leisure time had an equal or decreased risk of myocardial infarction compared with normal-weight but sedentary persons. In contrast, an increased risk was noted among the physically active obese persons (BMI ≥ 30).

The overall results, where increased risk is associated with overweight/obesity, and decreased risk is associated with leisure-time physical activity, as observed in this study, are in accordance with several other studies [2-6,11-13].

The joint effect of BMI and leisure-time physical activity, with equal or reduced risk of myocardial infarction among the overweight active persons compared with normal-weight sedentary persons corresponds to the results presented by Blair and Brodney [16]. Somewhat unexpectedly, we did not observe any reduced risk among the obese persons who were active, which is in contrast to others [16,20]. Instead, an increased risk of myocardial infarction was observed in this group.

However, there are studies where lack of an inverse relationship between physical activity and coronary heart disease in the highest BMI strata have been noted [21,22]. In some other studies, the active obese persons still had a higher cardiovascular or total mortality compared with inactive but normal-weight persons, irrespective of an inverse association between physical activity and mortality within the obese group [2,23].

It is known that obesity is associated with several adverse cardiovascular conditions, such as high blood pressure, and left ventricular hypertrophy [24,25]. Furthermore, some small studies have indicated that high BMI is associated with less efficient cardiac performance and higher blood pressure response during exercise, a higher level of oxidative stress after an exercise session, as well as a lower heart rate reserve [26-30]. It has also been shown that overweight subjects, even if they are regularly engaged in vigorous sport activities during leisure time, still have equal or lower heart rate variability at rest compared with sedentary lean subjects [31]. It may be hypothesized that intense exercise puts a very high strain on the cardiovascular system in obese subjects, which could result in an increased risk of acute myocardial infarction. Our results suggest that advising middle-aged or older individuals who are obese regarding physical training should be done with care, and that obese subjects should be encouraged to attain an initial weight reduction before taking up vigorous exercise.

### Study limitations and strengths

One advantage of the SHEEP study is that we have exposure information regarding recent leisure-time physical activity at the time prior to inclusion, as well as BMI at the time of inclusion in the study; this might explain some of the differences in our results compared with other studies. People might change both their BMI and physical activity level several times during life due to different reasons, and it is still unclear how different trajectories of the combination of BMI and physical activity relate to the risk of myocardial infarction.

In the analyses, we used the information regarding physical activity in the age interval to which the subject belonged at the time of the case occurrence. We also carried out analyses using information about physical activity in the age interval prior to inclusion in the study. The results were, however, unchanged.

We used data on height and weight from the clinical examination as the primary source of information regarding the calculation of BMI, and self-report of height and weight if data from the clinical examination were missing. For those where we had information from both the clinical examination and self-report, the BMI calculated from self-reported height and weight was closely correlated to the data from the clinical examination (BMI_clinical data _mean (std) 26.2 (3.9); BMI_self-report _mean (std) 25.9 (3.8); r = 0.92). Furthermore, when we restricted the analysis to the subjects for whom we had information on BMI from the clinical examination, the main results regarding the effect of leisure-time physical activity within the obese group did not change (data not shown).

Regarding the information about leisure-time physical activity/exercise we had to rely on self-report. This may lead to misclassification of the level of leisure-time physical activity. If the misclassification was non-differential between cases and controls, this would lead to estimated odds ratios closer to the null value than the actual true value, at least for the most active group when compared to the sedentary group. However, if the reporting of physical activity depended on disease status, the estimated odds ratios may be over- or underestimated. In general it might be expected that people tend to overestimate their physical activity level, due to social desirability. However, if the cases were less prone to do this and instead reported less activity compared with healthy controls, our estimated odds ratios regarding physical activity would be biased away from the null value. Since our overall results regarding leisure-time physical activity are in accordance with previous studies, including cohort studies where the problem of differential misclassification is unlikely to occur, we do not think that differential misclassification explains the major part of the findings regarding leisure-time physical activity in this study.

However, it has been shown that people in higher BMI strata are less precise in their reporting of leisure-time physical activity level than normal-weight people [32]. This might be part of the explanation for the weaker association between leisure-time physical activity and myocardial infarction among overweight and obese persons compared with normal-weight persons, observed in this study. This potential (non-differential) misclassification of physical activity is, however, unlikely to explain the opposite direction of the estimated relative risk among the most active obese persons when compared with the normal-weight or obese sedentary persons. Furthermore, we find it unlikely that obese cases should be more prone to overestimate previous physical activity level, compared with obese control subjects.

A possible explanation for the differences in the results when all cases vs. non-fatal cases only were included (apart from different quality in the proxy information for the fatal cases) could be a higher survival rate after a myocardial infarction for physically active persons, as has been reported by Wannamethee, et al [33].

The potential role of selection bias must be considered. In general, we had quite high response rates for both cases and controls (78 % for cases, 72 % for controls), which should prevent serious distortion of the results due to selection bias. However, the response rate was higher for non-fatal than for fatal cases (84 % and 62 %, respectively, with response for fatal cases provided by close relatives). Under the assumption that non-response was related to physical activity level (i.e. that low physical activity level was more common among non-respondents, and the proportion of non-respondents was higher among controls than non-fatal cases), we would have expected that the results from the analyses including non-fatal cases only would have yielded odds ratios at least equal to, or further away from the null value, compared with the odds ratios obtained from the analyses including all cases, if a substantial selection bias would have been present. This was not the case in our study. Furthermore, the main results for obesity and leisure-time physical activity are in agreement with several cohort studies, where selection bias is less likely to occur.

In some groups, e.g. in the group of lean subjects, the number of cases and controls were small. This is also the case when analyzing the combined effect of BMI and leisure-time physical activity in separate strata of men and women. The small number of cases and controls in these sub group analyses results in rather wide confidence intervals, which prevent any firm conclusions to be made from these analyses. However, the main results of the combined effect of BMI and leisure-time physical activity were similar in both men and women, and also in groups of low- and high socioeconomic status, in smokers and non-smokers, as well as in those below and above 60 years of age.

## Conclusion

In conclusion, leisure-time physical activity on a regular basis was associated with a decreased risk of myocardial infarction among the normal-weight persons. It also seemed to attenuate the increased risk associated with overweight. Somewhat unexpectedly, regular leisure-time physical activity did not seem to lower the risk of myocardial infarction among the obese persons in this study population. The reason for the lack of protection from physical activity among the obese is not clear, and needs to be explored further. It is possible, however, that the cardiovascular strain of physical exercise is more hazardous for the obese group. Therefore, if our results are correct, caution is advocated before advising obese individuals regarding physical training, and they should be encouraged to attain an initial weight reduction before taking up vigorous exercise.

## Abbreviations

BMI – Body Mass Index

CHD – Coronary Heart Disease

CI – Confidence Interval

MI – Myocardial Infarction

OR – Odds Ratio

The SHEEP study – The Stockholm Heart Epidemiology Program study

## Competing interests

The author(s) declare that they have no competing interests.

## Authors' contributions

EF contributed to the design of the present analysis of the SHEEP study, carried out the data analysis, interpreted the data and drafted the manuscript.

UdF contributed to the conception and design of the SHEEP study and revised the manuscript for important intellectual content.

AA contributed to the conception and design of the SHEEP study, and the acquisition of data, and revised the manuscript for important intellectual content.

CR contributed to the conception and design of the SHEEP study, and the acquisition of data, and revised the manuscript for important intellectual content.

JH contributed to the conception and design of the SHEEP study, and the acquisition of data, and revised the manuscript for important intellectual content.

LA contributed to the design of the present analysis of the SHEEP study, contributed substantially to the analysis and interpretation of the data, and revised the manuscript for important intellectual content.

All authors have read and approved the final manuscript.

## Pre-publication history

The pre-publication history for this paper can be accessed here:



## Figures and Tables

**Table 1:** Background characteristics of men and women, aged 45–70 years, in the Stockholm Heart Epidemiology Program Study, Stockholm, Sweden, 1992–1994.

	**Men**			**Women**		
	All cases	Non-fatal cases	Controls	All cases	Non-fatal cases	Controls

N	1204	968	1538	550	413	777
**Age **mean (SD)	59.0 (7.1)	58.5 (7.1)	59.2 (7.1)	62.0 (6.6)	61.6 (6.8)	62.0 (6.7)
**BMI **n (%)						
BMI < 20	34 (3)	22 (2)	42 (3)	50 (9)	25 (6)	37 (5)
20 ≤ BMI < 25	386 (33)	302 (31)	659 (43)	176 (33)	133 (32)	358 (46)
25 ≤ BMI < 30	558 (48)	486 (50)	675 (44)	179 (34)	148 (36)	264 (34)
BMI ≥ 30	193 (16)	155 (16)	162 (11)	124 (23)	105 (26)	117 (15)
*missing n*	*33*	*3*		*21*	*2*	*1*
**Leisure-time physical activity/exercise **n (%)						
Very little, occasional walks	670 (57)	512 (54)	663 (44)	363 (68)	249 (62)	374 (49)
Now and then, once per week	315 (27)	277 (29)	491 (32)	125 (24)	110 (27)	279 (36)
Twice per week or more	181 (16)	155 (16)	369 (24)	44 (8)	42 (10)	115 (15)
*missing n*	*38*	*24*	*15*	*18*	*12*	*9*
**Smoking **n (%)						
Current smoker	621 (52)	498 (52)	485 (32)	298 (54)	214 (52)	204 (26)
Non- or ex-smoker	581 (48)	469 (49)	1053 (68)	252 (46)	199 (48)	573 (74)
*missing n*	*2*	*1*				
**Socioeconomic status **n (%)						
Blue-collar	419 (35)	351 (36)	423 (28)	179 (33)	142 (34)	225 (29)
White-collar	722 (60)	588 (61)	1079 (70)	281 (51)	216 (52)	484 (62)
Other	63 (5)	29 (3)	36 (2)	90 (16)	55 (13)	68 (9)

**Table 2:** Body mass index (BMI) and leisure-time physical activity in relation to risk of acute myocardial infarction.

	**All cases**			**Non-fatal cases**		
	cases/controls	OR	95% CI	cases/controls	OR	95% CI

**Body Mass Index (BMI)**						
**All**						
BMI < 20^¶^	84/79	1.76	1.26–2.48	47/79	1.31	0.89–1.94
20 ≤ BMI < 25	562/1017	1*		435/1017	1	
25 ≤ BMI < 30	736/939	1.43	1.23–1.65	633/939	1.57	1.34–1.84
BMI ≥ 30	317/279	2.10	1.72–2.56	260/279	2.23	1.80–2.75
**Men**						
BMI < 20^†^	34/42	1.30	0.80–2.12	22/42	1.08	0.62–1.87
20 ≤ BMI < 25	386/659	1		302/659	1	
25 ≤ BMI < 30	557/675	1.44	1.21–1.72	485/675	1.58	1.31–1.90
BMI ≥ 30	193/162	2.07	1.61–2.67	155/162	2.08	1.59–2.72
**Women**						
BMI < 20^†^	50/37	2.29	1.39–3.76	25/37	1.55	0.87–2.76
20 ≤ BMI < 25	176/358	1		133/358	1	
25 ≤ BMI < 30	179/264	1.37	1.03–1.81	148/264	1.52	1.12–2.05
BMI 124/117 30	124/117	2.13	1.53–2.96	105/117	2.50	1.76–3.55
						
**Leisure-time physical activity**						
**All**						
Very little, occasional walks^¶^	1031/1037	1^#^		760/1037	1	
Now and then, once per week	440/770	0.64	0.55–0.74	387/770	0.74	0.63–0.87
Twice per week or more	225/484	0.54	0.45–0.65	197/484	0.62	0.51–0.76
**Men**						
Very little, occasional walks^†^	668/663	1		511/663	1	
Now and then, once per week	315/491	0.70	0.58–0.84	277/491	0.79	0.65–0.96
Twice per week or more	181/369	0.57	0.46–0.71	155/369	0.63	0.50–0.79
**Women**						
Very little, occasional walks^†^	363/374	1		249/374	1	
Now and then, once per week	125/279	0.52	0.40–0.68	110/279	0.64	0.48–0.86
Twice per week or more	44/115	0.44	0.30–0.65	42/115	0.58	0.39–0.87

OR-odds ratio; CI-confidence interval

* Normal-weight was used as reference category.

^# ^Being sedentary was used as reference category.

^¶ ^Adjusted for age, hospital catchment area, socioeconomic status, smoking and sex

^† ^Adjusted for age, hospital catchment area, socioeconomic status and smoking

**Table 3:** The combination of body mass index (BMI) and leisure-time physical activity/exercise in relation to risk of acute myocardial infarction. All cases and all controls.

	**BMI < 20**			**20 ≤ BMI < 25**			**25 ≤ BMI < 30**			**BMI ≥ 30**		
**Leisure-time physical activity/exercise**	cases/controls	OR	95% CI	cases/controls	OR	95% CI	cases/controls	OR	95% CI	cases/controls	OR	95% CI

**All**												
Very little, occasional walks^¶^	*57/42*	1.50	0.97–2.34	*326/394*	1*		*401/423*	1.19	0.96–1.46	*211/178*	1.55	1.20–2.01
Now and then, once per week	*18/24*	1.09	0.57–2.08	*141/343*	0.56	0.44–0.73	*214/330*	0.88	0.69–1.11	*63/73*	1.20	0.82–1.76
Twice per week or more	*6/13*	0.62	0.23–1.68	*79/263*	0.44	0.32–0.59	*103/181*	0.79	0.59–1.06	*35/27*	1.85	1.07–3.18
												
**Men**												
Very little, occasional walks^†^	*21/24*	0.88	0.47–1.66	*217/238*	1		*290/303*	1.13	0.88–1.45	*121/98*	1.47	1.05–2.05
Now and then, once per week	*9/10*	1.20	0.47–3.08	*96/206*	0.57	0.42–0.79	*165/232*	0.90	0.68–1.19	*41/43*	1.20	0.74–1.94
Twice per week or more	*3/8*	0.56	0.14–2.18	*61/203*	0.41	0.29–0.59	*90/138*	0.85	0.61–1.19	*25/20*	1.70	0.90–3.22
												
**Women**												
Very little, occasional walks^†^	*36/18*	2.40	1.25–4.62	*109/156*	1		*111/120*	1.32	0.91–1.93	*90/80*	1.65	1.09–2.48
Now and then, once per week	*9/14*	1.02	0.40–2.58	*45/137*	0.53	0.34–0.82	*49/98*	0.77	0.49–1.20	*22/30*	1.21	0.64–2.28
Twice per week or more	*3/5*	0.62	0.14–2.80	*18/60*	0.49	0.26–0.90	*13/43*	0.50	0.25–1.01	*10/7*	2.11	0.74–6.02

OR-odds ratio; CI-confidence interval

* The normal-weight (20 ≤ BMI < 25) and sedentary group was used as the reference category in all analyses.

^¶ ^Adjusted for age, hospital catchment area, socioeconomic status, smoking and sex

^† ^Adjusted for age, hospital catchment area, socioeconomic status and smoking

**Table 4:** The combination of body mass index (BMI) and leisure-time physical activity/exercise in relation to risk of acute myocardial infarction. Non-fatal cases and all controls.

	**BMI < 20**			**20 ≤ BMI < 25**			**25 ≤ BMI < 30**			**BMI ≥ 30**		
**Leisure-time physical activity/exercise**	cases/controls	OR	95% CI	cases/controls	OR	95% CI	cases/controls	OR	95% CI	cases/controls	OR	95% CI

**All**												
Very little, occasional walks^¶^	*29/42*	1.06	0.63–1.78	*236/394*	1*		*329/423*	1.33	1.06–1.67	*163/178*	1.65	1.25–2.17
Now and then, once per week	*12/24*	1.01	0.48–2.09	*121/343*	0.65	0.50–0.85	*194/330*	1.06	0.83–1.36	*60/73*	1.55	1.05–2.30
Twice per week or more	*4/13*	0.55	0.17–1.74	*65/263*	0.48	0.35–0.67	*95/181*	0.97	0.72–1.32	*32/27*	2.27	1.30–3.95
												
**Men**												
Very little, occasional walks^†^	*14/24*	0.78	0.39–1.59	*161/238*	1		*244/303*	1.25	0.96–1.64	*90/98*	1.42	0.99–2.04
Now and then, once per week	*7/10*	1.26	0.46–3.44	*83/206*	0.65	0.46–0.91	*149/232*	1.06	0.78–1.42	*38/43*	1.45	0.88–2.38
Twice per week or more	*1/8*	0.23	0.03–1.88	*48/203*	0.43	0.29–0.63	*82/138*	1.01	0.71–1.43	*23/20*	2.08	1.08–4.00
												
**Women**												
Very little, occasional walks^†^	*15/18*	1.47	0.67–3.19	*75/156*	1		*85/120*	1.49	0.99–2.26	*73/80*	2.01	1.29–3.14
Now and then, once per week	*5/14*	0.83	0.28–2.52	*38/137*	0.63	0.39–1.02	*45/98*	1.02	0.63–1.63	*22/30*	1.78	0.93–3.39
Twice per week or more	*3/5*	0.94	0.21–4.21	*17/60*	0.64	0.34–1.21	*13/43*	0.69	0.34–1.41	*9/7*	2.76	0.93–8.14

OR-odds ratio; CI-confidence interval

* The normal-weight (20 ≤ BMI < 25) and sedentary group was used as the reference category in all analyses.

^¶ ^Adjusted for age, hospital catchment area, socioeconomic status, smoking and sex

^† ^Adjusted for age, hospital catchment area, socioeconomic status and smoking

## References

[B1] Wannamethee G, Shaper AG, Walker M (2005). Overweight and obesity and weight change in middle aged men: impact on cardiovascular disease and diabetes. J Epidemiol Community Health.

[B2] Meyer HE, Søgaard AJ, Tverdal A, Selmer RM (2002). Body mass index and mortality: the influence of physical activity and smoking. Med Sci Sports Exerc.

[B3] Jonsson S, Hedblad B, Engström G, Nilsson P, Berglund G, Janzon L (2002). Influence of obesity on cardiovascular risk. Twenty-three-year follow-up of 22025 men from an urban Swedish population. Int J Obes.

[B4] Rosengren A, Wilhelmsen L, Lappasa G, Johansson S (2003). Body mass index, coronary heart disease and stroke in Swedish women. A prospective 19-year follow-up in the BEDA study. Eur J Cardiovasc Prevention Rehab.

[B5] Cho E, Manson JE, Stampfer MJ, Solomon CG, Colditz GA, Speizer FE, Willett WC, Hu FB (2002). A prospective study of obesity and risk of coronary heart disease among diabetic women. Diabetes Care.

[B6] Rosengren A, Wedel H, Wilhelmsen L (1999). Body weight and weight gain during adult life in men in relation to coronary heart disease and mortality. A prospective population study. Eur Heart J.

[B7] Manson JE, Hu FB, Rich-Edwards JW, Colditz GA, Stampfer MJ, Willett WC, Speizer FE, Hennekens CH (1999). A prospective study of walking as compared with vigorous exercise in the prevention of coronary heart disease in women. N Engl J Med.

[B8] Manson JE, Greenland P, LaCroix AZ, Stefanick ML, Mouton CP, Oberman A, Perri MG, Sheps DS, Pettinger MB, Sisovick DS (2002). Walking compared with vigorous exercise for the prevention of cardiovascular events in women. N Engl J Med.

[B9] Sesso HD, Paffenbarger RS, Lee IM (2000). Physical activity and coronary heart disease in men: the Harvard Alumni Health Study. Circulation.

[B10] Wagner A, Simon C, Evans A, Ferrieres J, Montaye M, Ducimetiere P, Arveiler D (2002). Physical activity and coronary event incidence in Northern Ireland and France; the Prospective Epidemiological Study of Myocardial Infarction (PRIME). Circulation.

[B11] Yusuf S, Hawken S, Ôunpuu S, Dans T, Avezuma A, Lanas F, McQueen M, Budaj A, Pais P, Varigos J (2004). Effect of potentially modifiable risk factors associated with myocardial infarction in 52 countries (the INTERHEART study): case-control study. Lancet.

[B12] Farahmand BY, Ahlbom A, Ekblom Ö, Ekblom B, Hallmarker U, Aronson D, Brobert GP (2003). Mortality amongst participants in Vasaloppet: a classical long distance ski race in Sweden. J Intern Med.

[B13] Eaton CB (1992). Relation of physical activity and cardiovascular fitness to coronary heart disease, part I: a meta-analysis of the independent relation of physical activity and coronary heart disease. J Am Board Fam Pract.

[B14] Martinez-Gonzales MA, Alfredo Martinez J, Hu FB, Gibney MJ, Kearney J (1999). Physical inactivity, sedentary lifestyle and obesity in the European Union. Int J Obes.

[B15] Thompson DL, Rakow J, Perdue SM (2004). Relationship between accumulated walking and body composition in middle-aged women. Med Sci Sports Exerc.

[B16] Blair SN, Brodney S (1999). Effects of physical inactivity and obesity on morbidity and mortality: current evidence and research issues. Med Sci Sports Exerc.

[B17] Fransson E, de Faire U, Ahlbom A, Reuterwall C, Hallqvist J, Alfredsson L (2004). The risk of acute myocardial infarction: interactions of types of physical activity. Epidemiology.

[B18] Reuterwall C, Hallqvist J, Ahlbom A, De Faire U, Diderichsen F, Hogstedt C, Pershagen G, Theorell T, Wiman B, Wolk A (1999). Higher relative, but lower absolute risks of myocardial infarction in women than in men: analysis of some major risk factors in the SHEEP study. J Int Med.

[B19] Hosmer DW, Lemeshow S (2000). Applied Logistic Regression.

[B20] Hu G, Tuomilehto J, Silventoinen K, Barengoc N, Jousilahti P (2004). Joint effects of physical activity, body mass index, waist circumference and waist-to-hip ratio with the risk of cardiovascular disease among middle-aged Finnish men and women. Eur Heart J.

[B21] Tanasescu M, Leitzmann MF, Rimm EB, Willett WC, Stampfer MJ, Hu FB (2002). Exercise type and intensity in relation to coronary heart disease in men. JAMA.

[B22] Dorn JP, Cerny FJ, Epstein LH, Naughton J, Vena JE, Winkelstein W, Schisterman E, Trevisian M (1999). Work and leisure time physical activity and mortality in men and women from a general population sample. Ann Epidemiol.

[B23] Hu FB, Willett WC, Li T, Stampfer MJ, Colditz GA, Manson JE (2004). Adiposity as compared with physical activity in predicting mortality among women. N Eng J Med.

[B24] Hubert HB (1986). The importance of obesity in the development of coronary risk factors and disease: the epidemiologic evidence. Ann Rev Public Health.

[B25] Kopelman PG (2000). Obesity as a medical problem. Nature.

[B26] Salvadori A, Fanari P, Fontana M, Buontemi L, Saezza A, Baudo S, Miserocchi G, Longhini E (1999). Oxygen uptake and cardiac performance in obese and normal subjects during exercise. Respiration.

[B27] Zanettini JO, Fuchs FD, Zanettini MT, Zanettini JP (2004). Is hypertensive response in treadmill testing better identified with correction for working capacity? A study with clinical, echocardiographic and ambulatory blood pressure correlates. Blood Pressure.

[B28] Tulio S, Eglé S, Greily B (1995). Blood pressure response to exercise of obese and lean hypertensive and normotensive male adolescents. J Hum Hypertens.

[B29] Vincent HK, Morgan JW, Vincent KR (2004). Obesity exacerbates oxidative stress levels after acute exercise. Med Sci Sports Exerc.

[B30] Ozcelik O, Aslan M, Ayar A, Kelestimur H (2004). Effects of body mass index on maximal work production capacity and aerobic fitness during incremental exercise. Physiol Res.

[B31] Rennie KL, Hemingway H, Kumari M, Brunner E, Malik M, Marmot M (2003). Effects of moderate and vigorous physical activity on heart rate variability in a British study of civil servants. Am J Epidemiol.

[B32] Norman A, Bellocco R, Bergström A, Wolk A (2001). Validity and reproducibility of self-reported total physical activity – differences by relative weight. Int J Obes Metab Disord.

[B33] Wannamethee G, Whincup PH, Shaper AG, Walker M, MacFarlane PW (1995). Factors determining case fatality in myocardial infarction "Who dies in a heart attack?". Br Heart J.

